# Efficient proton transport modelling for proton beam therapy and biological quantification

**DOI:** 10.1007/s00285-025-02212-1

**Published:** 2025-04-11

**Authors:** Ben S. Ashby, Veronika Chronholm, Daniel K. Hajnal, Alex Lukyanov, Katherine MacKenzie, Aaron Pim, Tristan Pryer

**Affiliations:** 1 Institute of Mathematical Innovation, University of Bath, Bath, UK; 2 Department of Mathematical Sciences, University of Bath, Bath, UK; 3 Department of Mathematics and Statistics, University of Reading, Reading, UK

**Keywords:** Proton transport, Bragg peak, Linear energy transfer, Relative biological effectiveness, Treatment planning, Radiotherapy modelling, 35L65, 35Q92, 92C50, 49M20, 65C05, 62P10

## Abstract

In this work, we present a fundamental mathematical model for proton transport, tailored to capture the key physical processes underpinning Proton Beam Therapy (PBT). The model provides a robust and computationally efficient framework for exploring various aspects of PBT, including dose delivery, linear energy transfer, treatment planning and the evaluation of relative biological effectiveness. Our findings highlight the potential of this model as a complementary tool to more complex and computationally intensive simulation techniques currently used in clinical practice.

## Introduction

**Fig. 1:**
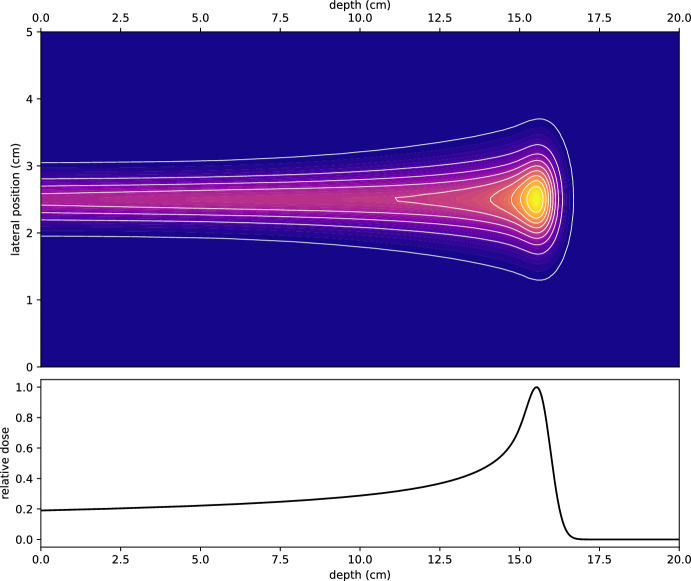
Simulated dose profile of a proton beam illustrating the Bragg peak. The simulation was performed using MCsquare with $1.21 \times 10^7$ particles, a beam width of 2 mm, without nuclear interactions. The initial proton energy is 150 MeV with a 1% energy spread, and the dose is integrated along the plane orthogonal to the beam axis

Proton Beam Therapy (PBT) has emerged as an important modality in the treatment of specific challenging cancers, particularly where conventional photon-based radiotherapy struggles to minimise irradiation to surrounding critical tissues. Pediatric cancers, skull base tumours, and complex head and neck malignancies are example cases where PBT offers a distinct advantage due to its ability to deposit energy with precision, peaking at the Bragg peak, see Fig. 1.

The Bragg peak enables precise targeting of tumours while sparing adjacent healthy tissue, making PBT an attractive option for complex clinical scenarios. However, despite its potential for superior dose profiles, there are fundamental challenges in treatment planning, verification, and uncertainty quantification. A key issue is the variability in patient anatomy during the treatment course, such as changes in water retention or tumour motion, which can lead to suboptimal tumour coverage or increased irradiation of healthy tissues. These challenges limit the potential of PBT to achieve superior therapeutic outcomes consistently.

Moreover, the intricacies of Relative Biological Effectiveness (RBE), which varies with proton energy and penetration depth, add a layer of complexity to treatment optimisation. The RBE, a measure of the relative damage caused by protons compared to photons, depends heavily on Linear Energy Transfer (LET), the rate at which energy is deposited along the proton track. Regions with higher LET are known to cause more complex and clustered DNA damage, which is harder for cells to repair, leading to increased biological effectiveness. This correlation makes LET a key metric for linking physical dose distributions to biological outcomes. As highlighted by Nystrom et al. (2020):

“We believe that the endless discussions of which is the most appropriate RBE-model and the exact values of the RBE for different tissue and in different parts of the dose distribution should be put on hold. Rather, efforts should be put in the development of clinically useful tools to visualise LET distributions and the possibility to include LET in the optimisation of proton treatment plans.”

This sentiment underscores the urgent need for practical, clinically-relevant methodologies to optimise PBT treatment plans while navigating biological complexities. In this work, we adopt a fundamental mathematical perspective to address some of these challenges in PBT treatment planning. Specifically, we focus on developing a robust and accessible framework for optimising dose distribution, incorporating considerations of RBE and its impact on tumour cell survival. A particular emphasis is placed on LET, enabling the integration of biological metrics into the optimisation process.

Our approach introduces:

- A simplified, yet precise, model that facilitates rapid exploration of treatment plan precision and its implications for therapeutic outcomes.
- A rigorous, accessible, mathematical formulation for optimising the biological effective dose, explicitly accounting for LET distributions and RBE variability.
- A biologically informed framework for interpreting treatment plans, incorporating cell survival fraction and RBE as key metrics.
- A detailed sensitivity analysis of the proposed framework, identifying the main parameters that influence treatment outcomes.

The goal is to provide tools that are not only theoretically sound but also practical for clinical implementation, bridging the gap between mathematical modelling and real-world PBT optimisation. This framework supports investigations towards personalised and adaptive therapy strategies, with the end goal of enhancing precision in challenging clinical scenarios by enabling the integration of spatially resolved biological metrics, such as LET, into treatment planning.

Modelling proton transport and dose distribution in PBT has traditionally relied on Monte Carlo simulations (Salvat 2013; Jabbari et al. 2014), which provide detailed physical insights but come with significant computational costs (Unkelbach et al. 2016). These methods remain the gold standard for accuracy but are impractical for real-time treatment planning. Recent advancements in neural network-based methodologies show promise for accelerating dose prediction by leveraging large datasets (Frizzelle et al. 2022; Fanou et al. 2023). However, these approaches are still nascent in clinical contexts and require extensive validation.

From a biological perspective, the role of RBE in treatment optimisation has been an important point of research. Unlike photon radiotherapy, where energy independence simplifies the calculation of biologically effective dose (BED) (Bellamy et al. 2015), proton radiotherapy demands careful calibration to account for RBE variability with depth and energy (Chaudhary et al. 2014). While multiple RBE models exist, the lack of consensus on their clinical applicability continues to pose challenges, and in practice virtually all treatment centres use a constant RBE of 1.1 (Gerweck et al. 1999; Underwood et al. 2016; Giantsoudi et al. 2013; Paganetti et al. 2019), despite the fact that there is broad consensus that RBE varies along the particle track, increasing near the Bragg peak (Hojo et al. 2017) to values significantly larger than 1.1. Indeed, RBE typically reaches values of approximately 1.6 in the falloff region of the Bragg peak (Paganetti 2014).

Finally, efforts to incorporate LET distributions into treatment planning are gaining traction (see (Unkelbach et al. 2016) for an example). These efforts align with the broader push toward integrating data-driven methodologies, such as neural networks, which have shown promise in accelerating dose prediction and enhancing planning workflows. Our framework complements such approaches by offering a rigorous and interpretable model for evaluating and validating biologically informed treatment plans, as well as providing tools to visualise the effect that varying optimisation routines to include additional quantities such as LET has upon the final plan.

It is worth noting that treatment planning is only one aspect of the broader radiotherapy workflow, which encompasses tumour growth modelling, temporal variations and interactions with other treatment modalities such as chemotherapy. While these dynamic aspects can be addressed using optimal control formulations (Schättler et al. 2015), this work focuses on stationary problems, providing a foundational framework that enables the capture of key physical and biological processes. In clinical practice, temporal changes such as tumour shrinkage or movement are addressed through adaptive radiotherapy, which relies on re-imaging, re-planning, or pre-optimised scenarios to ensure treatment quality across long, fractionated courses of therapy (Sonke et al. 2019).

In this context, the computational efficiency of our framework offers a significant advantage, particularly in adaptive workflows where rapid dose calculations and fast treatment plan optimisations are important. In this work we aim to bridge the gap between theoretical modelling and clinical applicability, laying the groundwork for examining biological metrics in current planning workflows.

The rest of this paper is structured as follows: in Sect. 2, we introduce the fundamental mathematical model, describing the principles of charged particle transport, stopping power, and LET and validate the model against existing Monte Carlo codes. In Sect. 3, we shift focus to biological metrics, exploring how the model can incorporate key measures such as the cell survival fraction and RBE to evaluate the biological effectiveness of proton beams. Section 4 addresses model uncertainties, providing a sensitivity analysis to quantify how variations in key parameters affect dose and LET predictions. Finally, Sect. 5 explores applications to treatment planning, demonstrating how the framework can optimise dose delivery and integrate biological metrics to enhance therapeutic outcomes.

## Modelling of charged particle transport

In this section we introduce fundamental modelling concepts in proton transport and discuss a fundamental model to aid in the exploration of the ideas discussed here.

**Fig. 2:**
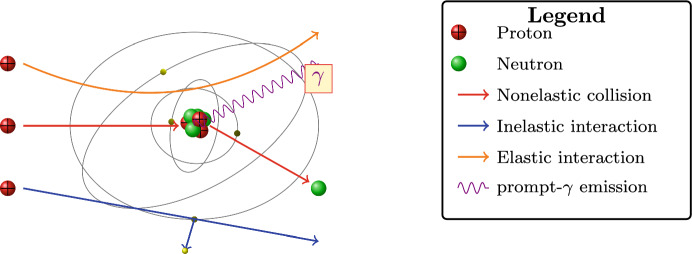
The three main interactions of a proton with matter. A *proton-nucleus collision, an**Coulomb interaction with atomic electrons and**Coulomb scattering with the nucleus*

### A model for proton transport

In this section, we introduce a simplified model for proton transport that builds upon the principles shown in Fig. 2. To that end, consider a bounded domain $X \subset {\mathbb {R}}^3$. For $0< E_{\min } < E_{\max }$, we define the interval $I = [E_{\min }, E_{\max }]$ as the set of admissible particle energies and let ${\mathbb {S}}^2$ represent the unit sphere, which describes possible particle trajectories.

At any given position $\vec {x} \in X$, with energy $E \in I$ and a trajectory direction $\vec {\omega } \in {\mathbb {S}}^2$, we are interested in modelling the particle fluence. The fluence, denoted $\psi (\vec {x}, E, \vec {\omega })$, describes the differential number of particles passing through a small surface area within an infinitesimal energy range. We let ${\mathscr {S}}$ denote the *stopping power*, defined in Sect. 2.3, as a function of the particle energy in a homogeneous domain.

To simplify the analysis, we make several assumptions regarding proton transport. First, we assume that *nonelastic collisions*, which involve nuclear interactions leading to secondary particle production, are rare relative to ionisation losses at therapeutic energies (50–250 MeV) and therefore do not significantly impact the fluence. Second, we assume that *angular scattering* is minimal. While multiple Coulomb scattering contributes to lateral beam broadening, small-angle deflections do not substantially alter the energy deposition profile along the primary trajectory, allowing us to model transport using straight-line motion. Lastly, we consider a *homogeneous medium*, which provides a first-order approximation to soft tissues. These assumptions lead to a transport model where the fluence satisfies

1 $$\begin{aligned} \vec {\omega } \cdot \nabla _{\vec {x}} \psi (\vec {x}, E, \vec {\omega }) + \dfrac{\partial }{\partial E} ({\mathscr {S}}(\vec {x}, E) \psi (\vec {x}, E, \vec {\omega })) = 0. \end{aligned}$$

This equation captures the balance between the particle’s motion through the medium and the energy loss through ionisation described by the stopping power.

Boundary conditions are required to close the model. The inflow condition specifies the fluence at the boundary of the domain

2 $$\begin{aligned} \partial X_-:= \{{\varvec{x}\in \partial X}: {\vec {n}(\vec {x}) \cdot \vec {\omega } < 0}\} \end{aligned}$$

where particles enter, while the energy cutoff condition ensures that no particles exist above the maximum energy, these read

3 $$\begin{aligned} \begin{aligned} \psi (\vec {x}, E, \vec {\omega })&= {\mathscr {G}}(\vec {x}, E, \vec {\omega }) \quad \,\forall \,\vec {x} \in \partial X_-, E\in I \\ \psi (\vec {x}, E_{\max }, \vec {\omega })&= 0 \quad \,\forall \,\vec {x} \in X, \vec {\omega } \in {\mathbb {S}}^2. \end{aligned} \end{aligned}$$

This formulation provides the foundation we use to explore proton transport.

**Fig. 3:**
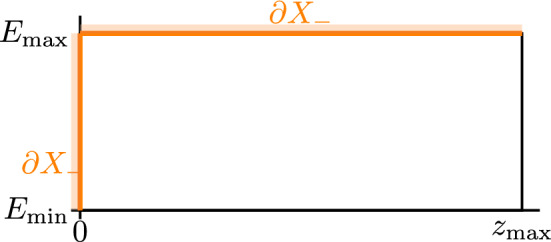
An illustration of the domain and the relevant inflow boundary

### Model simplification

Given the assumptions made, it is useful to consider the variable

4 $$\begin{aligned} z:= (\varvec{x} - \varvec{x}_0)\cdot \varvec{\omega }, \end{aligned}$$

where $\varvec{x}_0 \in \partial X_-$ is a given entrance point and $\varvec{\omega }\in {\mathbb {S}}^2$ is a given trajectory. We define

5 $$\begin{aligned} z_{\max }:= \inf \left\{ {s>0: ~ \varvec{x}_0 + s \varvec{\omega }\in \partial X, ~ \varvec{n}(\varvec{x}_0 + s \varvec{\omega }) \cdot \varvec{\omega }> 0}\right\} , \end{aligned}$$

which, for a given entrance point, represents the minimal path length to an outflow boundary. Then, for fixed $\varvec{x}_0$ and $\varvec{\omega }$, we define the one-dimensional stopping power and fluence in terms of the energy *E* and the one-dimensional track length *z* by the following change of variables,

6 $$\begin{aligned} \begin{aligned} S(z,E)&:= {\mathscr {S}}(\varvec{x}_0 + z \varvec{\omega }, E) \\ u(z,E)&:= \psi (\varvec{x}_0 + z \varvec{\omega }, E, \varvec{\omega }) \\ g(E)&:= {\mathscr {G}}(\varvec{x}_0, E, \varvec{\omega }). \end{aligned} \end{aligned}$$

We obtain the following problem for *u*:

7 $$\begin{aligned} \dfrac{\partial }{\partial z}u(z, E) + \dfrac{\partial }{\partial E}\!\left( {S(z, E)u(z, E)}\right) =0, \qquad \forall z,E \in (0, z_{\max })\times I, \end{aligned}$$

subject to the boundary conditions

8 $$\begin{aligned} \begin{aligned} u(0, E)&= g(E),~ \forall E \in I, \qquad \\ u(z,E_{\max })&= 0, ~ \forall z \in (0, z_{\max }). \end{aligned} \end{aligned}$$

Figure 3 gives a visualisation of the domain and inflow boundaries.

### Energy, range and stopping power

Suppose that a proton beam consisting of particles of a single energy $E_0$ enter a medium. There is a fundamental relationship between $E_0$ (in MeV) and the range $R_0$ the protons penetrate into the medium. For a homogeneous medium, this relationship is often modelled as a power law

9 $$\begin{aligned} R_0 = \alpha E_0^p, \end{aligned}$$

where $p \in [1,2]$ and $\alpha > 0$ are constants related to the mass density of the medium. Some indicative empirical values are given in Table 1.

**Table 1:** Range–energy relationship parameters for different media

Medium	*p*	$\alpha$
Water	$1.75 \pm 0.02$	$0.00246 \pm 0.00025$
Muscle	1.75	0.0021
Bone	1.77	0.0011
Lung	1.74	0.0033

Notice the parameter *p* remains relatively constant across different biological media. In contrast, the parameter $\alpha$ varies more significantly, as it is strongly dependent on the density and composition of each medium. The uncertainty in the water phantom is based on comparing three parameterisations of the Bragg Kleeman rule from (Pettersen 2018; Boon 1998; Bortfeld 1997)

From this relationship, one can derive a formula for the remaining energy *E*(*z*) at a given depth, $z \ge 0$ by observing that at depth *z*, the range of the beam is $R_0 - z$. Applying the range-energy relationship (9) at this depth yields

10 $$\begin{aligned} R_0 - z = \alpha E(z)^p, \end{aligned}$$

Solving for *E* we see

11 $$\begin{aligned} E(z) = \alpha ^{-\frac{1}{p}}\left( R_0 - z\right) ^{\frac{1}{p}}. \end{aligned}$$

This expression describes the energy of the proton beam as a function of depth. The linear stopping power, defined as the energy loss per unit distance travelled, can then be computed by

12 $$\begin{aligned} S(z):= -\frac{\,\textrm{d}E(z)}{ \,\textrm{d}z} = \frac{\alpha ^{-\frac{1}{p}}}{p}\left( R_0 - z\right) ^{\frac{1}{p} - 1}. \end{aligned}$$

Finally, since the relationship (11) is invertible for $0 \le z \le R_0$, the stopping power can be expressed in terms of energy:

13 $$\begin{aligned} S(E) = \frac{1}{\alpha p}E^{1-p}. \end{aligned}$$

This representation, called the Bragg–Kleeman rule, illustrates how the stopping power decreases as a function of energy which is the property that yields the forward facing peaked nature of the Bragg peak.

#### Remark 2.4

(Relativistic effects) Proton therapy typically uses proton energies in the range of 50 to 250 MeV. While these energies are high enough to require accurate modeling of stopping power, they are not so high that relativistic effects dominate. In this intermediate energy range, the Bragg–Kleeman model provides a sufficiently accurate approximation of stopping power while maintaining simplicity and computational efficiency (Ulmer 2007).

**Fig. 4:**
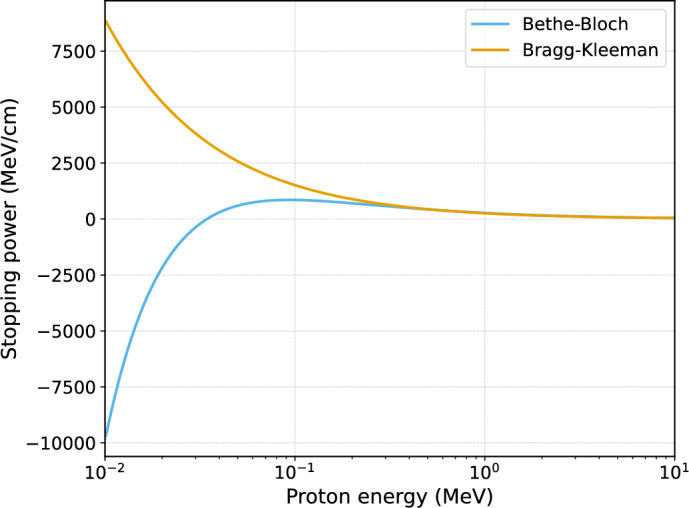
Stopping power as a function of energy for the Bragg–Kleeman and Bethe–Bloch models. The two models show good agreement in the intermediate energy range relevant to proton therapy however differ dramatically in the low energy range

The Bethe–Bloch formula, illustrated in Fig. 4, is more precise at relativistic speeds as it incorporates corrections and additional parameters necessary at very high energies (Navas 2024). However, it also introduces complexity to calculations and exhibits unphysical behaviour at low energy levels, making it less suitable for the energy ranges used in proton therapy.

#### Remark 2.5

(Heterogeneous tissue) In this work, we focus on a homogeneous medium specifically a water phantom, in which case $\alpha$ and *p* can be treated as constant. However, in heterogeneous tissues, density and composition vary with depth, leading to discontinuities in the stopping power. This affects the range-energy relationship, as the parameters are no longer constant but instead piecewise defined according to the material properties of each region. In such cases, the stopping power can be modelled as a piecewise function to account for different tissue types (Cox et al. 2024).

### Closed form solution

Due to the form of the stopping power, the equation is hyperbolic in nature, we can therefore use the method of characteristics to construct a closed form solution. To that end, the characteristic curves satisfy

14 $$\begin{aligned} E^p = E_{\max }^p - \frac{z}{\alpha }. \end{aligned}$$

This curve represents the energy trajectory of a monoenergetic proton beam with initial energy $E_{\max }$ in the (*z*, *E*)-plane. Integrating (7) along these characteristic curves, the solution for fluence *u*(*z*, *E*) is

15 $$\begin{aligned} \begin{aligned} u(z, E)&= \!\left( {E^p + \frac{z}{\alpha }}\right) ^{\frac{1-p}{p}} g\!\left( {\!\left( {E^p + \frac{z}{\alpha }}\right) ^{\frac{1}{p}}}\right) E^{p-1}. \end{aligned} \end{aligned}$$

### Computation of absorbed dose

The absorbed dose, *D*(*z*), represents the energy deposited per unit mass at a given depth and can be calculated by integrating the stopping power weighted by the particle fluence over the energy range

16 $$\begin{aligned} \begin{aligned} D(z) =&\int _{E_{\text {min}}}^{E_{\text {max}}}\dfrac{S(E)}{\rho (z)} u(z,E) \,\textrm{d}E \\ =&\frac{1}{\alpha p}\frac{1}{\rho (z)} \int _{E_{\text {min}}}^{E_{\text {max}}} \!\left( {E^p + \frac{z}{\alpha } }\right) ^{\frac{1-p}{p}} g\!\left( {\!\left( {E^p + \frac{z}{\alpha }}\right) ^{\frac{1}{p}}}\right) \,\textrm{d}E. \end{aligned} \end{aligned}$$

This formulation captures the full spectrum of proton energies within *I*, accounting for their interactions with the medium and the resulting energy deposition.

**Fig. 5:**
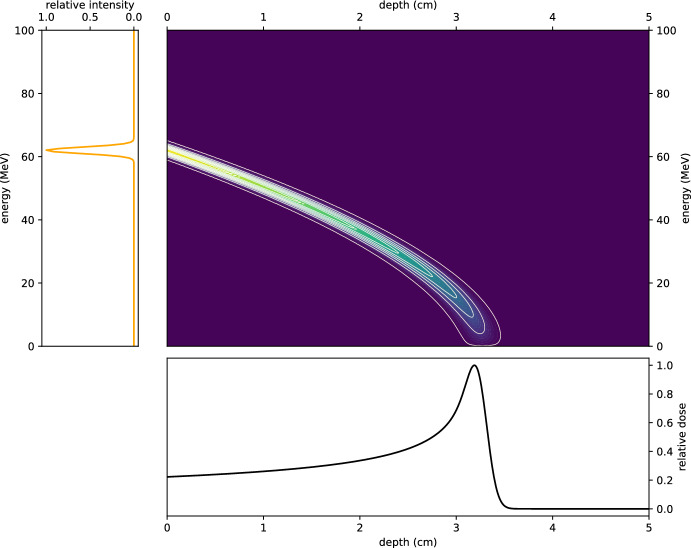
A visualisation of dose calculation. Left: the initial Gaussian energy profile of a 62 MeV proton beam with a 1% energy spread. Middle: the fluence in depth-energy space, illustrating how the beam evolves as it travels through the medium. Bottom: the resultant dose as a function of depth

To provide an intuitive understanding of this setup, Fig. 5 presents a visualisation of the fluence and resulting dose for a 62 MeV proton beam with a 1% energy spread. The figure consists of a grid of panels illustrating three components:

- The initial energy distribution *g*(*E*) of the proton beam is shown on the left, highlighting the Gaussian profile centred at 62 MeV.
- The middle panel depicts the fluence in depth-energy space, demonstrating how the protons’ energy evolves along their trajectories as they penetrate the medium.
- The bottom panel shows the resulting dose *D*(*z*), plotted as a function of depth, capturing the energy deposited within the target region.

This visualisation effectively connects the initial beam properties to the resulting dose distribution within the context of (16).

### Linear energy transfer

A related concept to stopping power is the Linear Energy Transfer (LET), which describes the energy locally absorbed by the medium per unit distance. The physical definition from the ICRU (Thomas 2012) defines LET, or the restricted linear electronic stopping power as

17 $$\begin{aligned} L_{\Delta }= \frac{\,\textrm{d}E_{\Delta }}{\,\textrm{d}z}, \end{aligned}$$

which represents the energy lost $\,\textrm{d}E_{\Delta }$ by the primary charged particle in interactions with electrons, along a distance $\,\textrm{d}x$ minus energy carried away by energetic secondary electrons having initial kinetic energies greater than $\Delta$. In the limit $\Delta \rightarrow \infty$, LET is equivalent to the stopping power, this is referred to as the unrestricted LET. Essentially, stopping power accounts for the total energy loss, while LET focuses on the energy absorbed locally in the medium, which will be important in understanding the biological impact of radiation later in this work.

If we assume that all of the energy lost is absorbed locally by the material, then we have two notions of calculating the average LET from the particle fluence: track-averaged and dose-averaged (Kalholm 2021).

#### Track-averaged LET

18 $$\begin{aligned} L_T(z) = \frac{\int _E u(z, E) S(E) \,\textrm{d}E}{\int _E u(z, E) \,\textrm{d}E}. \end{aligned}$$

#### Dose-averaged LET

19 $$\begin{aligned} L_D(z) = \frac{\int _E u(z, E) S^2(E) \,\textrm{d}E}{\int _E u(z, E)S(E) \,\textrm{d}E}. \end{aligned}$$

##### Remark 2.9

(Monoenergetic simplification) The track-averaged LET is sometimes referred to as fluence-averaged or particle-averaged LET. Track- and dose-averaged LET are defined for a polyenergetic beam, where *g*(*E*) is an arbitrary distribution of energies. However, in the case of a truly monoenergetic beam, where

20 $$\begin{aligned} g(E) = c \delta (E - E_0) \end{aligned}$$

for some constant *c* and initial energy $E_0 \in {\mathbb {R}}$, the expressions for track-averaged and dose-averaged LET simplify to

21 $$\begin{aligned} L_T(z) = L_D(z) = S\!\left( { \!\left( {E_0^p - \frac{z}{\alpha }}\right) ^{\frac{1}{p}} }\right) . \end{aligned}$$

### Comparison with Monte Carlo codes

This section compares the analytical model described above with established simulation tools to validate its performance against key benchmarks. Specifically, we evaluate whether the simplified model, given by Eq. 7, can accurately reproduce both qualitatively correct and quantitatively reasonable behaviour. The comparison is conducted against the Monte Carlo simulation tool MCsquare (Souris 2016) and the Geant4-based TOPAS framework (Faddegon 2020).

To assess the accuracy of the one-dimensional analytical model, we consider a pristine Bragg peak simulation as a computational benchmark. A mono-energetic proton beam with an initial energy of $E_0 = 62 \, \text {MeV}$ and a fluence of $1.21 \, \text {gigaprotons/cm}^2$ is used as the input beam. The analytical model employs standard Bragg–Kleeman parameter values for water ($\alpha = 2.2 \times 10^{-3}$, $p = 1.77$) as reported in Bortfeld (1997), while the Monte Carlo codes use their respective default material parameters for water. To account for the energy spread in the Monte Carlo simulations, the standard deviation of the proton energy spectrum is set to $\epsilon E_0$, with $\epsilon = 0.01$.

The boundary condition for the analytical model is defined as:

22 $$\begin{aligned} u(0, E) = 1.21 \times 10^9 \times C \exp \!\left( {-\frac{(E - E_0)^2}{2 \epsilon ^2 E_0^2}}\right) , \end{aligned}$$

where *C* is a normalisation constant ensuring that the integral of the spectrum matches the total fluence of $1.21 \, \text {gigaprotons/cm}^2$.

For the Monte Carlo codes, a three-dimensional water phantom is simulated and the depth-dose curve is obtained by integrating the dose over the plane perpendicular to the beam axis. Depth-dose curves for the analytical model are computed using Eq. 16.

**Fig. 6:**
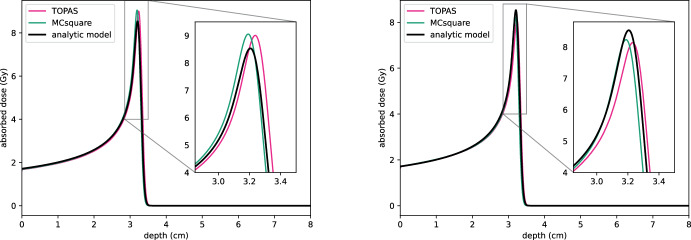
Comparison of depth-dose curves for a 62 MeV mono-energetic proton beam in water obtained from the one-dimensional analytical model (black), MCsquare (green) and TOPAS (pink). Left: nuclear interactions are excluded in the Monte Carlo simulations. Right: nuclear interactions are included. All simulations are scaled such that the total number of incoming protons is $1.21 \, \text {gigaprotons}$, ensuring a consistent comparison of absorbed dose

The comparison results are shown in Fig. 6, where close agreement between the models can be observed. The left panel shows results excluding nuclear interactions in the Monte Carlo simulations, while the right panel includes these effects. Both cases demonstrate that the analytical model captures the depth-dose behaviour with high fidelity, providing a computationally efficient alternative to Monte Carlo simulations.

It is important to note that the analytic model represents a substantial simplification compared to Monte Carlo methods, relying on the Bragg–Kleeman formulation and neglecting relativistic effects. While some variation in agreement is observed in Fig. 6, the difference is minimal—one case leads to a slight overestimation of dose, while the other results in a slight underestimation. More significantly, the two Monte Carlo codes themselves exhibit discrepancies, illustrating that their deviation from one another is comparable to their deviation from the analytic model. This highlights the variability in dose prediction, even among detailed transport methods.

## Biological metrics

Treatment planning can be thought of as translating a physician’s prescription into a set of parameters that define the radiotherapy delivered to a patient (Kooy 2015). The treatment plan should be ‘optimal’ in some sense, as defined in Sect. 5. Typically, the goal is to deliver a dose that closely matches a prescribed target dose profile using a suitable metric. Absorbed dose is widely used for this purpose because it is measurable and can be computed accurately in numerical simulations. In addition, prescriptions from clinicians are given as a dose due to the long history of use and gathered expertise in photon based radiotherapy. However, dose is only a proxy for biological effect and there is no simple one-to-one relationship between absorbed dose and biological outcomes (Nystrom et al. 2020). For example, LET and cell type must also be considered, and for this reason, metrics beyond dose have been incorporated into carbon ion beam treatment planning (where LET plays a larger role than for protons) for over two decades (Kanai et al. 1999; Inaniwa 2015; Karger et al. 2017). In this section, we focus on cell survival rates as a biological metric to assess treatment plan quality and introduce the concept of the relative biological effectiveness (RBE) for proton beams, as well as the biological dose (BD).

To formalise these ideas, specific examples of biological effect must be considered. The choice of metric should align with the desired clinical endpoints and be measurable or detectable (Karger et al. 2017). Examples of such endpoints include tumour control probability (TCP) and normal tissue complication probability (NTCP), which are often estimated from in vivo experiments. For simplicity, we focus on the cell survival fraction, a quantity measurable in vitro via clonogenic assay (Serrano-Mendioroz et al. 2023). A cell is considered ‘killed’ or inactive if it is unable to proliferate. This metric provides an objective measure of treatment efficacy while avoiding the complexities of more comprehensive metrics like TCP. However, while the survival fraction is relevant for tumour control, it may not be an ideal measure for toxicity in healthy tissues (Nystrom et al. 2020), which is an important consideration in treatment planning.

For X-rays, the survival fraction $\mathcal{S}\mathcal{F}_{\text {X-ray}}$ is accurately modelled in vitro as a function of absorbed dose $D_{\text {X-ray}}(x)$ using the *linear-quadratic model* (Kellerer 1978):

23 $$\begin{aligned} \mathcal{S}\mathcal{F}_{\text {X-ray}}(z; D_{\text {X-ray}}) = \exp \left( -c_{\text {X-ray}}(z) D_{\text {X-ray}}(z) - \beta _{\text {X-ray}}(z) D_{\text {X-ray}}(z)^2 \right) , \end{aligned}$$

where $c_{\text {X-ray}}(z)$^1^ and $\beta _{\text {X-ray}}(z)$ are model parameters typically estimated through regression, with their dependence on cell species captured via their spatial variation. We note that the linear quadratic model becomes less accurate in some regimes (Hanin 2010), but is effective in many practical dose ranges.

### Remark 3.1

(Interpretation of the parameters in the linear-quadratic model) As described in Chadwick et al. (1973), the parameters in the linear-quadratic model have physical interpretations. Cell death following irradiation is primarily caused by DNA double-strand breaks, either from a single particle interaction or from two single-strand breaks created by separate interactions. The parameters $c_{\text {X-ray}}(z)$ and $\beta _{\text {X-ray}}(z)$ correspond to the expected number of single and double-strand breaks per unit dose, respectively. While a general derivation of the model is given in Kellerer (1978), the parameters are often determined empirically by fitting the model to experimental data.

### Linear-quadratic model for protons

Equation (23) implies a one-to-one relationship between absorbed dose and cell killing. Predicting cell survival following irradiation with proton beams is more complex than for X-rays due to the additional dependence on LET. This dependence can be incorporated into the linear-quadratic (LQ) framework and many studies have investigated how LET affects the parameters of the LQ model (Hawkins 1998; Goodhead et al. 1992; Belli et al. 1993). LET-dependent models remain widely used and well-studied (Wilkens et al. 2004; Chaudhary et al. 2014).

Experimental evidence suggests that, within clinically relevant LET ranges, the coefficient *c* depends approximately linearly on LET:

24 $$\begin{aligned} c(z; L_D) = c_{0}(z) + \lambda (z) L_D(z), \end{aligned}$$

where $c_0(z)$ corresponds to the value of *c* for X-rays and $\lambda (z)$ represents the tissue-dependent LET sensitivity. A more detailed model in Tilly (2002) (see also (Carabe et al. 2012)) proposes modifying this dependence based on the ratio of $c_{\text {X-ray}}$ to $\beta _{\text {X-ray}}$, introducing exponential dependence when this ratio is large. However, for simplicity, we assume $\lambda (z)$ is constant for a given tissue type.

The relationship between $\beta$ and LET is less clear and constant $\beta$ is commonly assumed (Chaudhary et al. 2014). Nevertheless, some studies, such as (Carabe-Fernandez et al. 2007), have examined potential LET-dependent variations in $\beta$.

Following Chaudhary et al. (2014), we set $c_0 = c_{\text {X-ray}}$ in Eq. 24. The spatial variation of $c_{\text {X-ray}}$, $\beta$ and $\lambda$ reflects tissue-specific responses to radiation. Table 2 summarises these parameters for two cell types: AG01522 (skin cells) and U87 (malignant brain tumour cells) (Chaudhary et al. 2014).

**Table 2:** Parameters for the LET-dependent LQ model (25), measured for two cell types (Chaudhary et al. 2014)

Cell type	$c_{\text {X-ray}}$ ($\text {Gy}^{-1}$)	$\lambda$ ($\mu \text {m}\, \text {keV}^{-1}\, \text {Gy}^{-1}$)	$\beta$ ($\text {Gy}^{-2}$)
AG01522 (skin)	$0.54 \pm 0.06$	0.0451	$0.051 \pm 0.038$
U87 (brain tumour)	$0.11 \pm 0.028$	0.0127	$0.059 \pm 0.024$

The survival fraction of cells irradiated by a proton dose *D* and LET $L_D$ is then given by:

25 $$\begin{aligned} \mathcal{S}\mathcal{F}(z; D, L_D) = \exp \left( -c(z; L_D) \cdot D(z) - \beta (z) D(z)^2\right) . \end{aligned}$$

**Fig. 7:**
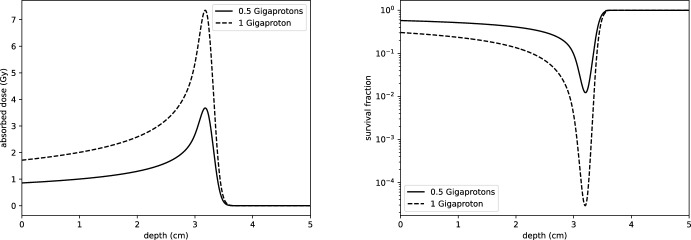
Left: depth-dose profiles for 62 MeV proton beams of two different intensities, Right: corresponding survival fractions of cells against depth assuming a homogeneous medium of cells, computed using the model (25)

For a given dose profile, *D*(*z*), the surviving fraction of cells at each depth *z* may be predicted by the model (25). An example for a Bragg peak is shown in Fig. 7. It is worth noting that this model may require further modification for heavier charged particles such as carbon ions (Wilkens et al. 2004).

### Relative biological effectiveness

Relative Biological Effectiveness (RBE) is an important metric in radiobiology, defined as the ratio of doses required from two radiation sources to achieve the same biological effect. It captures differences in energy deposition and clinical outcomes between radiation modalities (Paganetti et al. 2019). Here, we consider RBE in terms of cell survival fraction.

Proton beams differ from X-rays in their energy deposition characteristics. In particular, low-energy protons near the distal end of their range inflict greater biological damage on tissue. In Hojo et al. (2017), an increase in molecular markers of double-strand DNA breaks ($\gamma$H2AX foci) is observed at the distal end of the Bragg peak, consistent with Remark 3.1. It is well known that the RBE of protons exceeds 1.0 and a constant value of 1.1 has been widely adopted in clinical practice (Gerweck et al. 1999; Underwood et al. 2016; Giantsoudi et al. 2013; Paganetti et al. 2019). While some studies support this approximation, others suggest it is insufficient (Tilly 2002).

Quantifying RBE is important to fully exploit the advantages of proton therapy. However, this task is challenging due to complex dependencies on biological factors, including cell type, cell cycle phase (Underwood et al. 2016) and clinical endpoint (Paganetti et al. 2019), as well as limited data for many tissues (Grassberger et al. 2011). Results from in vitro experiments on specific cell lines often do not generalise to others due to significant biological variation. Additionally, RBE depends on dose and radiation quality, typically characterised by LET (Wilkens et al. 2004).

In this work, we consider a spatially variable notion of relative biological effectiveness as follows. Given a depth dose curve *D*(*z*) and LET profile $L_D(z)$, at each point *z* a surviving fraction of cells may be computed as described above. The equivalent photon dose, i.e. the dose that yields the same cell survival fraction, is found by inverting the functional relationship given in Eq. (23). Specifically, given *D*, we seek $D_{\text {X-ray}}$ such that:

26 $$\begin{aligned} \mathcal{S}\mathcal{F}(z; D(z), L_D(z)) = \mathcal{S}\mathcal{F}_{\text {X-ray}}(z; D_{\text {X-ray}}(z)) \end{aligned}$$

for all *z*. We may then define a spatially variable relative biological effectiveness as

27 $$\begin{aligned} RBE(z, D, L_D):= \frac{D_{\text {X-ray}}(z)}{D(z)}. \end{aligned}$$

**Fig. 8:**
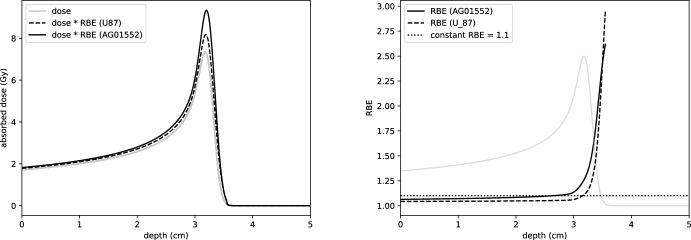
Left: RBE-weighted dose curves for a 62 MeV mono-energetic proton beam in water phantom, calculated using the TDRA model for cell survival and parameters from Chaudhary et al. (2014) for AG01522 and U87 cell lines. Right: Corresponding RBE. Dose curve illustrates RBE behaviour along the Bragg peak and is not to scale

The RBE-weighted dose is then defined to be the product of dose and RBE, i.e. precisely the equivalent X-ray dose $D_{\text {X-ray}}$. This quantity can be used to compare the biological effect with photon dose curves. RBE and RBE-weighted dose curves are shown in Fig. 8 for two different cell types studied in Chaudhary et al. (2014). We observe that the RBE is in line with the clinical value of 1.1 up until the Bragg peak, but becomes significantly larger in the distal falloff region.

### Biological dose (BD)

Unfortunately, optimising for dose delivery that results in a given survival fraction is problematic for a number of reasons. Discussion of these issues is postponed until Sect. 5. In this section an alternative is presented which is more amenable to optimisation.

A natural alternative is to consider the logarithm of the survival fraction, as is common in log-likelihood maximisation. This idea appears in Unkelbach et al. (2016); McIntyre et al. (2023), where the LET-weighted dose, often referred to as the biological dose (BD), is used as an optimisation metric. In [Unkelbach et al. (2016), Appendix A], a discussion of a simpler linear exponential model for the survival fraction is used is given. It can be viewed as a simplification of the linear quadratic model for which fractionation effects are neglected. The model is given by:

28 $$\begin{aligned} \mathcal{S}\mathcal{F}_{\text {lin}}(z; D(z), L_D(z)):= \exp \left( - c(z; L_D) D(z)\right) . \end{aligned}$$

The biological dose is then defined as

29 $$\begin{aligned} BD(z):= - \log (\mathcal{S}\mathcal{F}_{\text {lin}}(z; D(z), L_D(z)))/ c_{\text {X-ray}} = D(z) \left( 1 + \frac{\lambda }{c_{\text {X-ray}}} L_D(z)\right) , \nonumber \\ \end{aligned}$$

where *D*(*z*) is the absorbed dose, $L_D(z)$ is the dose-averaged LET, and $\lambda / c_{\text {X-ray}}$ quantifies the contribution of LET to the biological effect (cf. Eqs. (24) & (25)). This formulation balances physical dose delivery with biological considerations, making it a promising metric for treatment planning.

## Model uncertainties and sensitivity analysis

In this section, we investigate how uncertainty in the stopping power parameters $\alpha$ and *p* influences the predicted dose distribution. Given that stopping power governs the range-energy relationship, variations in these parameters can impact the Bragg peak position and overall dose deposition. While in realistic biological tissues, $\alpha$ would vary discontinuously with depth due to differences in tissue composition, we focus here on an idealised homogeneous water medium to isolate and quantify the effects of parametric uncertainty in a controlled setting. This provides a baseline for assessing how uncertainty propagates through the model, independent of the additional complexities introduced by tissue heterogeneity. The empirical values for $\alpha$ and *p* used in this study are derived from experimental measurements in water phantoms, which are appropriate for this homogeneous case but may require modification for heterogeneous media, as indicated in Remark Remark 2.5.

With this motivation in mind, this section aims to quantify the uncertainty in the magnitude and position of *D*(*z*) (16) when the stopping power parameters $\alpha$ and *p* from the Bragg–Kleeman rule in Eq. 13 are uncertain. Specifically, we consider the dose as parameterised by $\alpha$ and *p*, such that $D(z) = D(z;\alpha ,p)$. Two methods, an active subspace approach and a Monte Carlo simulation, are implemented to analyse the impact of uncertainty in the model.

### Active subspace method

We apply an active subspace method to evaluate the relative importance of the parameters $\alpha$ and *p* over specified ranges. This approach involves examining the dose across the phase space of $\alpha$ and *p* at a fixed point in the domain. The direction perpendicular to the contour lines in this space indicates the path along which the greatest change in dose occurs, providing insight into the relative sensitivities of $D(z;\alpha ,p)$ to $\alpha$ and *p*. Furthermore, the orientation of these contour lines is orthogonal to the eigenvectors of the covariance matrix at that point (see (Sullivan 2015, §10.5) for further details).

The parameters $\alpha$ and *p* are modelled as independent normally distributed random variables, with means $(\mu _\alpha , \mu _p)$ and variances $(\sigma _\alpha , \sigma _p)$:

30 $$\begin{aligned} \begin{aligned} \alpha&\sim {\mathcal {N}}(\mu _\alpha , \sigma _\alpha ) \\ p&\sim {\mathcal {N}}(\mu _p, \sigma _p). \end{aligned} \end{aligned}$$

For all simulations, we take $\mu _\alpha = 0.00246$ and $\mu _p = 1.75$, as shown in Table 1.

We consider three cases for the standard deviations $\sigma _\alpha$ and $\sigma _p$: absolute values, relative values, and empirical estimates based on data. For the absolute case, we set $\sigma _\alpha = \sigma _p = 0.0001$. For the relative case, $\sigma _\alpha$ and $\sigma _p$ are taken to be 1% of their respective mean values. For the empirical case, we use the data from Table 1, assuming the 95% confidence intervals represent twice the standard deviation. Thus, $\sigma _\alpha$ and $\sigma _p$ are scaled by 1/1.96 to align the normal distribution with these intervals. Table 3 summarises these choices.

**Table 3:** Standard deviations $\sigma _\alpha$ and $\sigma _p$ for the three case studies

Case	$\sigma _\alpha$	$\sigma _p$
Absolute	0.0001	0.0001
Relative	0.0000246	0.0175
Empirical	0.000128	0.0102

**Fig. 9:**
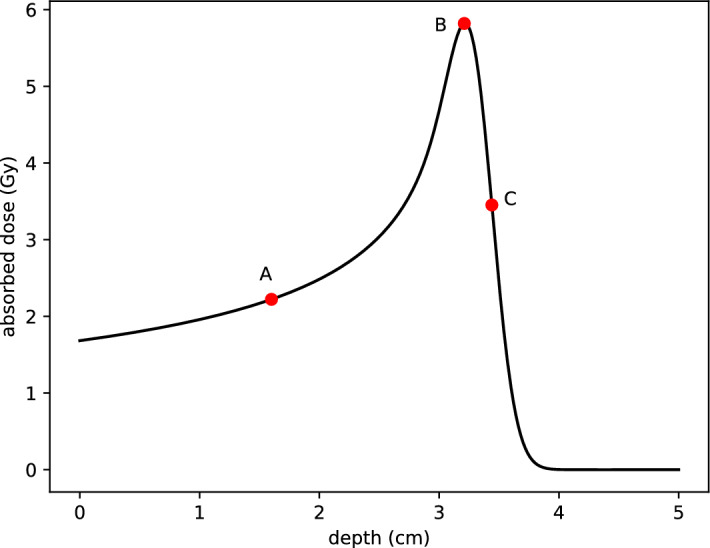
Positions for the active subspace analysis. Point *A* is halfway between the start and the Bragg peak, point *B* is at the peak, and point *C* is at the position with the steepest gradient. The initial beam has an energy of 62 MeV, with a spread of $5\%$

A sensitivity analysis is conducted at three points in the domain, labelled *A*, *B*, and *C*, as shown in Fig. 9. Point *A* lies midway between the start of the beam and the Bragg peak, point *B* is at the Bragg peak, and point *C* is at the location of the steepest gradient.

**Fig. 10:**
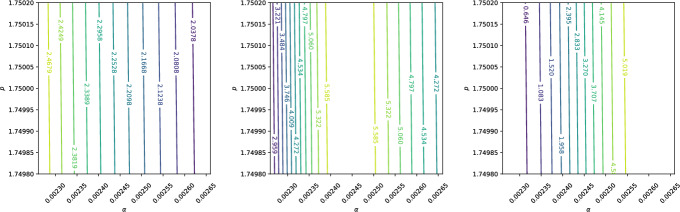
Contour plot of dose at points A, B, and C when $\alpha$ and *p* are assumed to follow normal distributions with absolute standard deviations $\sigma _\alpha = \sigma _p = 0.0001$

**Fig. 11:**
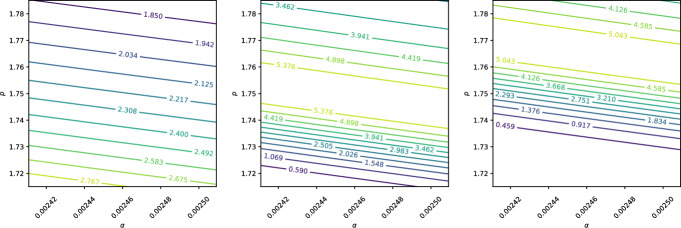
Contour plot of dose at points A, B, and C when $\alpha$ and *p* are assumed to follow normal distributions with relative standard deviations $\sigma _\alpha = 0.01\mu _\alpha = 0.0175$ and $\sigma _p = 0.01\mu _p = 0.0000246$

**Fig. 12:**
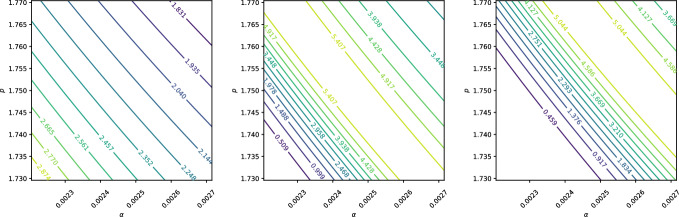
Contour plot of dose at points A, B, and C when $\alpha$ and *p* are assumed to follow normal distributions with empirical standard deviations $\sigma _\alpha = 0.000128$ and $\sigma _p = 0.0102$

Contour plots illustrating the sensitivities at points *A*, *B*, and *C* are shown in Figs. 10, 11, and 12, respectively. The *x*- and *y*-axes of these figures represent the ranges $(\mu _\alpha - 2\sigma _\alpha , \mu _\alpha + 2\sigma _\alpha )$ and $(\mu _p - 2\sigma _p, \mu _p + 2\sigma _p)$, ensuring that $\alpha$ and *p* span their 95% confidence intervals.

Figure 10 illustrates that, when absolute uncertainties are considered, the contour lines are nearly vertical, indicating that $D(x;\alpha , p)$ is significantly more sensitive to changes in $\alpha$ than to equivalent changes in *p*. In contrast, Fig. 11 shows that when relative uncertainties are used, a 1% change in *p* has a greater impact on $D(x;\alpha , p)$ than a 1% change in $\alpha$. Finally, Fig. 12, which uses empirical standard deviations based on Table 1, demonstrates that $D(x;\alpha , p)$ is equally sensitive to both parameters over the examined phase plane.

It is important to note that while Fig. 10 highlights $\alpha$’s greater influence under absolute uncertainties and Fig. 11 shows *p*’s dominance under relative uncertainties, Fig. 12 reflects a balance in sensitivity when using empirically motivated standard deviations. This motivates the inclusion of uncertainty in both $\alpha$ and *p* in the subsequent Monte Carlo analysis.

### Monte Carlo simulation

In this section, we investigate how uncertainty in the stopping power parameters $\alpha$ and *p* affects the shape and position of the dose curve $D(z;\alpha ,p)$. Using Monte Carlo simulations, we quantify the overall uncertainty in $D(z;\alpha ,p)$ as well as the uncertainty in the depth of the Bragg peak. As in Sect. 4.1, we model $\alpha$ and *p* as independent, normally distributed random variables with means $(\mu _{\alpha },\mu _p)$ and standard deviations $(\sigma _{\alpha },\sigma _p)$. Specifically, we take $\mu _{\alpha } = 0.00246$ and $\mu _p = 1.75$, and use empirical standard deviations $\sigma _{\alpha } = 0.000128$ and $\sigma _p = 0.0102$, corresponding to $95\%$ confidence intervals.

The nominal dose curve, computed with $\alpha = \mu _{\alpha }$ and $p = \mu _p$, is denoted as $D^*(z;\mu _{\alpha },\mu _p)$ and serves as the “true” reference dose curve. This curve corresponds to the assumed parameter values $\mu _{\alpha }=0.00246$ and $\mu _{p}=1.75$, as presented in Table 1. In the figures that follow, $D^*(z;\mu _{\alpha },\mu _p)$ is included to illustrate how uncertainties in $\alpha$ and *p* influence the dose curve.

It is important to note that the nominal dose curve $D^*(z;\mu _{\alpha },\mu _p)$ is not equivalent to the mean dose curve, as $D^*(z;{\mathbb {E}}[\alpha ],{\mathbb {E}}[p]) \ne {\mathbb {E}}[D(z,\alpha ,p)]$. As a result, the nominal dose curve does not necessarily lie within the calculated confidence intervals, which reflect the distribution of $D(z;\alpha ,p)$. This underscores the impact of parameter uncertainty on the dose curve and highlights the importance of considering the full range of variability in $\alpha$ and *p*.

**Fig. 13:**
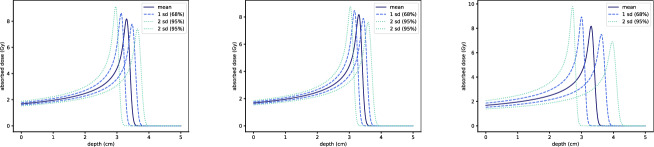
Sensitivity of $D(z;\alpha ,p)$ to under- and overestimation of $\alpha$ and *p* by one or two standard deviations. The nominal dose curve $D^*(z;\mu _{\alpha },\mu _p)$ is shown for reference. On the left, only $\alpha$ has been under- or overestimated; in the centre only *p*; and on the right both $\alpha$ and *p*

In Fig. 13, we show how the dose curve changes when $\alpha$ and *p* deviate by one or two standard deviations in either the positive or negative direction. The results indicate that deviations in either $\alpha$ or *p* alone induce moderate changes in $D(z;\alpha ,p)$. However, simultaneous deviations in both parameters cause larger changes in both the magnitude and the location of the Bragg peak.

Notably, the probability of both $\alpha$ and *p* being off by $2\sigma$ simultaneously is significantly lower than the probability of a single parameter deviating by $2\sigma$, assuming independence. For instance, under the normal distribution assumption, the probability of $\alpha$ being off by $2\sigma _{\alpha }$ is $5\%$, while the probability of simultaneous $2\sigma$ deviations for both parameters is $0.25\%$.

**Fig. 14:**
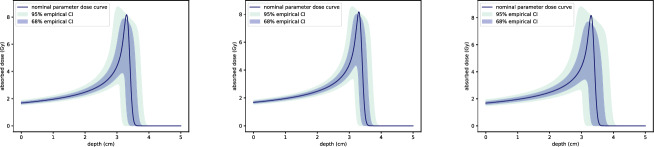
Confidence intervals for the variation in dose $D(z;\alpha ,p)$ when $\alpha$ and *p* are normally distributed with means $\mu _{\alpha } = 0.00246, \mu _p = 1.75$ and standard deviations $\sigma _{\alpha } = 0.000128, \sigma _p = 0.0102$. The nominal dose curve $D(z;\mu _{\alpha },\mu _p)$, resulting from the assumed parameter values $\mu _{\alpha }$ and $\mu _p$, is plotted in dark blue. The $68\%$ and $95\%$ confidence intervals are shown as shaded regions, estimated using 25,000 independent random samples of $\alpha$ and *p*. On the left, only uncertainty in $\alpha$ has been included; in the centre only uncertainty in *p*, and on the right uncertainty in both $\alpha$ and *p* (color figure online)

Figure 14 presents the estimated $68\%$ and $95\%$ confidence intervals for the dose curve $D(z;\alpha ,p)$. These intervals are derived using a Monte Carlo approach, with 25,000 independent random samples of $\alpha$ and *p*. Empirical quantiles are computed at each depth *z* to generate the ensemble of dose curves.

The results show that introducing uncertainty in either $\alpha$ or *p* individually leads to similar confidence intervals. However, when uncertainty is included for both parameters simultaneously, the confidence intervals for the dose curve become significantly larger. This demonstrates that the combined uncertainties in $\alpha$ and *p* amplify the overall uncertainty in dose deposition, emphasising the importance of accurately characterising both parameters.

**Fig. 15:**
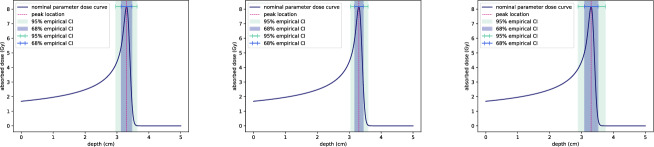
Confidence intervals for the variation in peak depth $z_{\text {peak}}$ when $\alpha$ and *p* are normally distributed with means $\mu _{\alpha } = 0.00246, \mu _p = 1.75$ and standard deviations $\sigma _{\alpha } = 0.000128, \sigma _p = 0.0102$. The nominal dose curve $D(z;\mu _{\alpha },\mu _p)$, resulting from the assumed parameter values $\mu _{\alpha }$ and $\mu _p$, is plotted in dark blue. The corresponding nominal peak depth of the curve $D(z;\mu _\alpha ,\mu _p)$ is plotted as a dashed red line. The $68\%$ and $95\%$ confidence intervals for $z_{\text {peak}}$ are shown as shaded regions, estimated using 25,000 independent random samples of $\alpha$ and *p*. On the left, only uncertainty in $\alpha$ has been included; in the centre only uncertainty in *p*, and on the right uncertainty in both $\alpha$ and *p* (color figure online)

In Fig. 15, we show the estimated $68\%$ and $95\%$ confidence intervals for the peak position $z_{\text {peak}}$ of the dose curve. These intervals are also derived using a Monte Carlo approach with 25,000 independent random samples of $\alpha$ and *p*.

The results indicate that the confidence intervals for $z_{\text {peak}}$ are comparable in magnitude when uncertainty is included for either $\alpha$ or *p* alone. However, when both parameters are simultaneously uncertain, the confidence intervals for $z_{\text {peak}}$ are considerably larger, consistent with the shifts in $z_{\text {peak}}$ observed in Fig. 13. Even relatively small uncertainties in $\alpha$ and *p* result in a $95\%$ confidence interval for $z_{\text {peak}}$ of approximately $\pm 1$ cm, a level of uncertainty significant for treatment planning. This highlights the need for precise parameter estimation to ensure accurate dose delivery.

## Treatment planning

To complete our study, we now examine how the model developed can be applied to treatment planning in proton therapy.

The goal of treatment planning is to determine the optimal initial beam angles, intensities, and energies such that the dose delivered to a cancerous region is maximised while minimising the biological damage to surrounding healthy tissues. This objective can be framed as a control problem.

Efficient optimisation methods are crucial in Intensity-Modulated Proton Therapy (IMPT), where practitioners often generate multiple treatment plans along an approximate Pareto surface to balance competing objectives and select the most suitable plan for the patient. Furthermore, the optimisation of proton therapy treatment plans involves a significantly larger number of decision parameters compared to Intensity-Modulated Radiation Therapy (IMRT) Chen (2010).

### Dose optimisation as a constrained least squares problem

We consider the spatial domain divided into disjoint regions of healthy and tumourous tissues, denoted $\Omega _H$ and $\Omega _T$, respectively. For simplicity, we assume $\Omega _T = [z_{\text {prox}}, z_{\text {dist}}]$. Given a target dose profile *T*(*x*), the objective is to construct an input beam $g: [E_{\text {min}}, E_{\text {max}}] \rightarrow {\mathbb {R}}$ such that the resulting dose *D*(*z*) closely approximates *T*(*z*).

To approach this problem, we require:

- A discrete representation of the input beam *g*;
- A forward model $g \rightarrow D$ to predict the dose profile;
- A metric to quantify the difference between *D*(*z*) and *T*(*z*).

To represent the input beam, we assume it is a superposition of a finite set of Gaussian-shaped basis beams, $\varphi _i$, $i = 1, \ldots , N_y$, each centred at a principal energy $E_i$ with variance $\sigma _i^2$. This formulation reduces the space of possible input functions *g* to a finite-dimensional vector space spanned by the basis beams:

31 $$\begin{aligned} g(\vec {y}, E) = \sum _{i=1}^{N_y} y_i \varphi _i(E), \end{aligned}$$

where $\vec {y} = (y_1, \ldots , y_{N_y})$ represents the weights or intensities of each constituent beam. Each $\varphi _i$ approximates a mono-energetic beam, and the choice of Gaussian-shaped beams ensures practical feasibility given equipment constraints, as discussed in Markman (2002). Alternative representations, such as piecewise constant or linear approximations, may lead to input beams that are difficult to realise in practice.

The forward model is provided by the one-dimensional analytical model from Sect. 2, which is linear with respect to the initial beam energy *g*. This allows the total dose profile to be expressed as a linear combination of precomputed dose profiles $D_i(z)$ corresponding to unit-intensity beams:

32 $$\begin{aligned} D(\vec {y}, z) = \sum _{i=1}^{N_y} y_i D_i(z). \end{aligned}$$

By precomputing the dose profiles $D_i(z)$ for each basis beam $\varphi _i$, the optimisation problem is reduced to finding the optimal coefficients $\vec {y}$, which is computationally efficient.

To measure how well $D(\vec {y}, z)$ approximates *T*(*z*), we define a cost function that penalises deviations between the dose and the target. Let *w*(*z*) be a non-negative weighting function, then the cost functional is

33 $$\begin{aligned} l(\vec {y}):= \int _X w(z)\left( D(\vec {y}, z) - T(z)\right) ^2 \, \,\textrm{d}x. \end{aligned}$$

For practical implementation, we evaluate the dose at a finite set of points $z_0, \ldots , z_{N_x}$ and approximate the integral using the composite trapezoidal rule. This leads to the discrete cost functional

34 $$\begin{aligned} L(\vec {y}):= \sum _{j=1}^{N_x} \frac{1}{2} w(z_j)\left( D(\vec {y}, z_j) - T(z_j)\right) ^2 (z_j - z_{j-1}). \end{aligned}$$

#### Definition 5.2

(Treatment planning optimisation problem) Given beams $\varphi _1, \ldots , \varphi _{N_y}$, target and weighting functions *T*, *w*, and an admissible set *Y*, find $\vec {y} \in Y$ such that:

35 $$\begin{aligned} L(\vec {y}) \le L(\vec {y}') \quad \forall \vec {y}' \in Y. \end{aligned}$$

#### Remark 5.3

(Weighting function *w*) The weighting function *w* provides flexibility in defining the cost function, allowing different priorities in the treatment plan. We illustrate this with the following examples:

1. In the simplest case, where the goal is to deliver a specified dose to a target region $\Omega _T$ with no restrictions elsewhere, *w* can be set as the indicator function of $\Omega _T$:
  36 $$\begin{aligned} w = \mathbb {1}_{\Omega _T}. \end{aligned}$$
2. When additional considerations, such as sparing an organ at risk (OAR) within $\Omega _O \subseteq \Omega _H$, are required, the weighting function can assign different priorities to regions. For example:
  37 $$\begin{aligned} w(z) = {\left\{ \begin{array}{ll} w_T & \text {for } z \in \Omega _T, \\ w_O & \text {for } z \in \Omega _O, \\ w_H & \text {otherwise}. \end{array}\right. } \end{aligned}$$
  Here, setting $w_O \gg w_T$ reflects a higher priority for sparing the OAR over achieving the target dose in the tumour.

#### Remark 5.4

(Admissible set *Y*) The admissible set *Y* allows the inclusion of practical constraints on beam intensities. For instance, beams must have non-negative intensity, so *Y* must satisfy:

38 $$\begin{aligned} Y \subseteq \{\vec {y} \in {\mathbb {R}}^{N_y}: y_i \ge 0 \,\, \forall i\}. \end{aligned}$$

Additionally, upper bounds on intensity may be enforced due to equipment limitations or safety constraints.

#### Selection of the inflow energy profiles

**Fig. 16:**
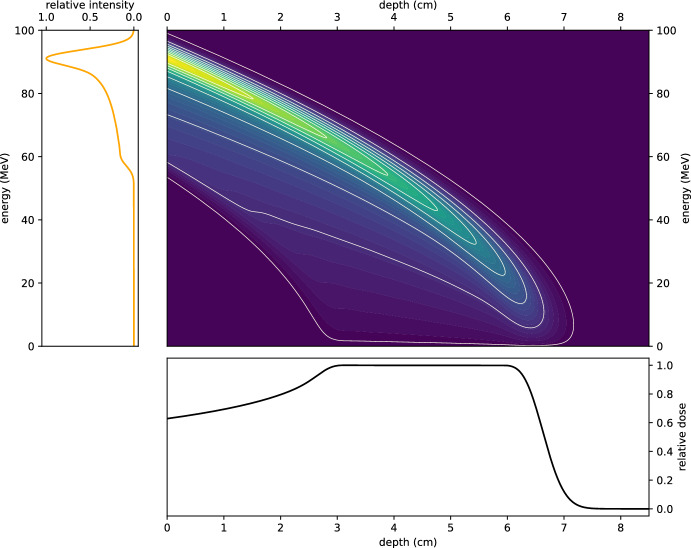
Visualisation of the input beam, fluence, and dose profile for Example 1. Left: the optimised input beam intensities across different energies. Middle: the fluence in depth-energy space, showing how the superposition of beams evolves through the medium. Right: the resulting dose profile as a function of depth, achieving a uniform dose within the target region $\Omega _T$

The success of the optimisation problem in 5.2 depends on appropriate choices for the beam profiles $\varphi _i$ and the admissible set *Y*. Physically, it is desirable for the spread-out Bragg peak (SOBP) to cover the tumour region $\Omega _T$. This requires the ranges of the constituent beams $\varphi _i$ to lie within $\Omega _T$ (Fig. 16).

The range of a proton beam, determined by the Bragg–Kleeman rule (see Eq. (9)), can be inverted to compute the energy *E* of protons with a given range *R*:

39 $$\begin{aligned} E = \left( \frac{R}{\alpha }\right) ^{1/p}. \end{aligned}$$

This relationship allows the selection of principal energies (the centres of the Gaussian profiles for each beam) such that the ranges satisfy:

40 $$\begin{aligned} z_{\text {prox}} \le \alpha E_i^p \le z_{\text {dist}}. \end{aligned}$$

**Fig. 17:**
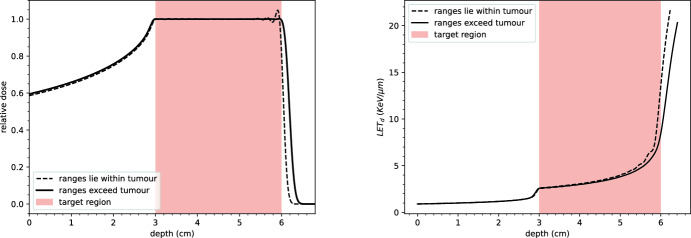
Spread-out Bragg peaks (left) and dose-averaged LET curves (right) resulting from the optimisation problem 5.2. Dashed lines: beam ranges are equally spaced between $z_{\text {prox}}$ and $z_{\text {dist}}$. Solid lines: beam ranges are equally spaced between $z_{\text {prox}}$ and $z_{\text {dist}} + 0.15$. Allowing slight extension of ranges into healthy tissue improves dose uniformity within the tumour

The choice of beam principal energies and widths significantly affects the appearance of the resulting SOBP. As illustrated in Fig. 17, restricting all beam ranges to lie strictly within the tumour region can lead to oscillations at the distal end of the SOBP. These oscillations persist even when the boundary condition is resolved with a greater number of beams. Allowing some beams with energies $E_i$ such that $R(E_i) > z_{\text {dist}}$ reduces these oscillations but increases the dose delivered to the surrounding healthy tissues.

#### Example 1: uniform dose delivery

We let $\Omega _T = [3, 6]$ and $\Omega _H = X \backslash \Omega _T$. The weighting function is chosen as described in Eq. 37, with $w_T = 1$ and $w_H = 0$. For this example, we set $N_y = 30$, with beam energies $E_i$ selected such that their ranges are equally spaced in $[z_{\text {prox}}, z_{\text {dist}} + \frac{1}{4}]$, and set $\sigma _i^2 = 1$ for all *i*. The admissible set *Y* is defined as:

41 $$\begin{aligned} Y = \{\vec {y} \in {\mathbb {R}}^{N_y}: y_i \ge 0 \,\, \forall i\}, \end{aligned}$$

to enforce non-negativity of beam intensities.

In practice, it is typical to deliver a homogeneous dose to the tumour region (Grassberger et al. 2011). As a first numerical experiment, we define the target dose profile as:

42 $$\begin{aligned} T(z) = {\left\{ \begin{array}{ll} 1 & \text {for } z \in \Omega _T, \\ 0 & \text {otherwise}, \end{array}\right. } \end{aligned}$$

and aim to find parameters $\vec {y}$ such that:

43 $$\begin{aligned} \Vert w(x)^{1 / 2}\left( D(\vec {y}, z) - T(z)\right) \Vert _{L^2} \rightarrow \min . \end{aligned}$$

**Fig. 18:**
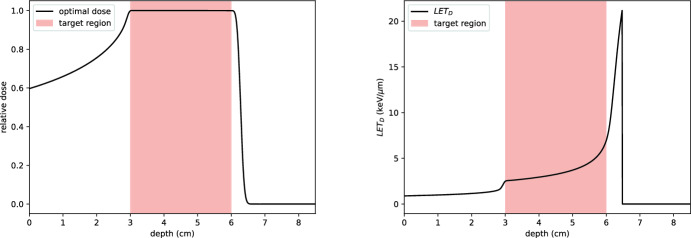
Left: dose profile resulting from the optimisation in Example 1. Right: corresponding dose-averaged LET. A total of 30 energy levels are used, with energies chosen such that their ranges are equally spaced and cover the target region. Uniform dose delivery to the tumour is achieved

The optimisation problem is solved using the Broyden–Fletcher–Goldfarb–Shanno (BFGS) algorithm. Results are shown in Fig. 18. The solution achieves highly uniform coverage of the target region. Specifically, if $\vec {y}^*$ is the optimal set of parameters, the relative error satisfies:

44 $$\begin{aligned} \frac{L(\vec {y}^*)}{L((0,0,\ldots ,0))} \approx 10^{-6}, \end{aligned}$$

where:

45 $$\begin{aligned} L((0,0,\ldots ,0)) = \int _X w(z) T(z)^2 \, \textrm{d} z. \end{aligned}$$

To provide a visual representation of the optimisation process and the resulting dose distribution, Fig. 16 illustrates the input beam configuration, the fluence in depth-energy space, and the final dose profile for Example 1. This figure parallels the visualisation provided earlier for the pristine Bragg peak (see Fig. 5), extending to the optimised spread-out Bragg peak (SOBP) used in this treatment plan. This visualisation connects the optimised input beam parameters to the resulting dose distribution.

#### Example 2: organ at risk (OAR)

In this example, we retain the tumour region $\Omega _T = [3, 6]$ but introduce an organ at risk (OAR) in $\Omega _O = [6, 8]$. The weighting function *w* is modified to penalise dose delivered to the OAR, and is defined as:

46 $$\begin{aligned} w(z) = {\left\{ \begin{array}{ll} w_T = 1 & \text {for } z \in \Omega _T, \\ w_O = 10 & \text {for } z \in \Omega _O, \\ w_H = 0 & \text {otherwise}. \end{array}\right. } \end{aligned}$$

As in Example 1, we set $N_y = 30$ and select beam energies such that their ranges are equally spaced in $[z_{\text {prox}}, z_{\text {dist}} + \frac{1}{4}]$ (Fig. 18).

**Fig. 19:**
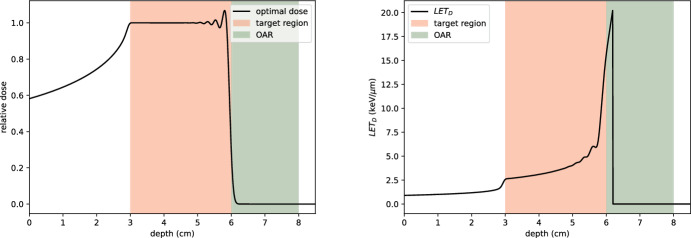
Left: dose profile resulting from the optimisation in Example 2. Right: corresponding dose-averaged LET. Penalising dose in the OAR (shaded green) reduces dose penetration into healthy tissue but introduces slight under-dosing and oscillation in the tumour’s distal region (color figure online)

The results of this optimisation are shown in Fig. 19. The competing objectives of delivering sufficient dose to the tumour while sparing the OAR result in less uniform dose coverage within the tumour region. Compared to Fig. 18, there is a notable reduction in LET near the distal edge of the tumour, accompanied by a significant decrease in dose delivered to the OAR. However, this optimisation introduces oscillations and slight under-dosing in the tumour’s distal region, which may be clinically relevant depending on the treatment context.

**Fig. 20:**
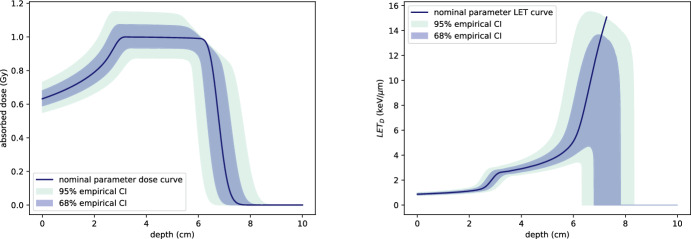
Confidence intervals for a spread-out Bragg peak and the corresponding dose-averaged LET. The confidence intervals are obtained as in Sect. 4, by introducing uncertainty in the stopping power parameters $\alpha$ and *p*, and estimating the empirical confidence intervals from 25,000 independent samples using Monte Carlo methods. The dark blue curves represent the nominal dose and LET profiles, calculated using the assumed parameter values $\mu _{\alpha }$ and $\mu _p$

To further understand the implications of model uncertainty, we examine the effects of parameter variability on the spread-out Bragg peak (SOBP) for a scenario that includes an OAR. Figure 20 shows the Monte Carlo-estimated confidence intervals for both the SOBP and the corresponding dose-averaged LET. The methodology follows that described in Sect. 4, where uncertainty in the stopping power parameters $\alpha$ and *p* is introduced, and empirical confidence intervals are computed from 25,000 independent samples.

The results highlight significant uncertainty in the maximum dose attained within the SOBP, as well as in the falloff region beyond the tumour. Similarly, there is substantial uncertainty in LET, particularly near the depth where the LET curve becomes sharply peaked. These uncertainties are clinically important to consider in treatment planning, as they heavily affect the balance between tumour coverage and OAR sparing. When an OAR is located directly behind the tumour, such uncertainties can compound the challenge of achieving an optimal treatment plan.

### Optimisation based on biological metrics

An alternative approach to treatment planning involves prescribing the fraction of surviving cells as a function of space, aligning the optimisation process more closely with biological outcomes. For instance, one could aim to kill 90% of cells within the tumour, resulting in an objective function of the form:

47 $$\begin{aligned} L_{\mathcal{S}\mathcal{F}}(\vec {y}):= \sum _{j=1}^{N_x}\frac{1}{2} \left( w(z)(\mathcal{S}\mathcal{F}(\vec {y}, z) - T_{\mathcal{S}\mathcal{F}}(z))^2\right) (z_j - z_{j-1}), \end{aligned}$$

where $\mathcal{S}\mathcal{F}(\vec {y}, x)$ denotes the survival fraction and $T_{\mathcal{S}\mathcal{F}}(x)$ represents the target survival fraction.

While this approach is biologically motivated, it presents practical challenges. Due to the exponential relationship between dose and survival fraction, the optimisation problem becomes more computationally expensive and can sometimes yield counterintuitive results. For instance, regions receiving excessive dose may not incur a significant penalty in the objective function, as the survival fraction in those regions is already near zero.

To address these limitations, a biologically weighted dose metric is often preferred. By incorporating biological weighting factors into the dose, the optimisation problem becomes linear in terms of the control parameters, significantly reducing computational cost. Moreover, this approach avoids the spurious results associated with excessive dose regions, providing a more robust framework for treatment planning while retaining a biologically informed perspective.

#### Example 3: uniform LET-weighted dose

In this example, we optimise for a uniform biological dose within the target region $\Omega _T$, defined as:

48 $$\begin{aligned} T_{BD}(z):= {\left\{ \begin{array}{ll} 1 & \text {for } z \in \Omega _T, \\ 0 & \text {otherwise}. \end{array}\right. } \end{aligned}$$

**Fig. 21:**
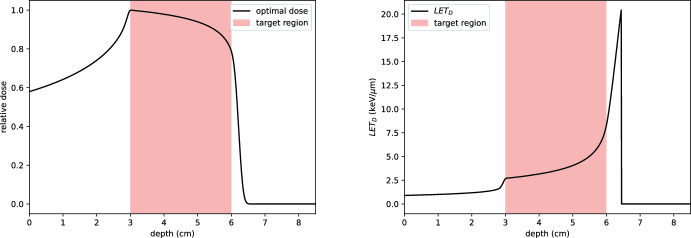
Left: dose profile resulting from the optimisation in Example 3. Right: corresponding dose-averaged LET. A total of 30 energy levels are used, with energies chosen such that their ranges are equally spaced and cover the target region

The input beams are chosen as in Examples 1 and 2, with the weight function *w* set to $\mathbb {1}_{\Omega _T}$. The results of this optimisation are shown in Fig. 21. Optimising for biological dose introduces a trade-off: some dose conformity is sacrificed at the distal part of the tumour to account for the higher LET values that occur there. Consequently, the dose delivered is significantly lower than that obtained by considering absorbed dose alone, reflecting the heightened biological impact of protons near the Bragg peak.

**Fig. 22:**
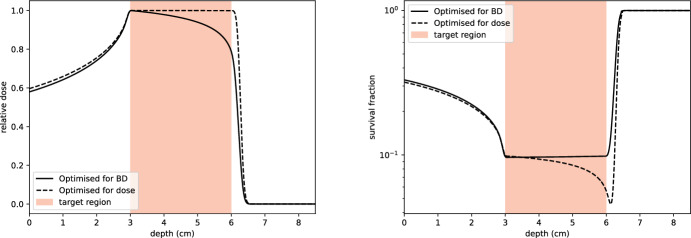
Comparison of relative dose (left) and survival fraction profiles (right) resulting from optimisation for absorbed dose (dashed line) and biological dose (solid line). The survival fraction was computed using the linear-quadratic model, with the $\alpha$ parameter accounting for LET, for AG01522 cells using parameters from (Chaudhary et al. 2014). Optimising for biological dose results in a tapered dose profile at the distal end of the target region, delivering significantly less dose to healthy tissue while maintaining an almost constant biological effect within the target

Interestingly, the largest biological effect, computed using the linear-quadratic model, is observed outside the target region when LET is taken into account, as shown in Fig. 22. This arises because the LET-weighted optimisation naturally prioritises regions of higher biological effectiveness, even if they fall outside the prescribed dose boundaries. A comparison of the survival fraction profiles resulting from absorbed dose and biological dose optimisation demonstrates this effect. The tapering of the dose profile towards the distal end of the target region ensures significantly less dose is delivered to healthy tissue, while maintaining a near-uniform biological effect within the tumour.

It is instructive to compare these results with those in Fig. 4 of (Giovannini 2016), where a similar shape is observed in RBE-weighted dose curves. The survival fraction profiles in Fig. 22 exhibit a comparable trend, underscoring the alignment between LET-weighted dose optimisation and RBE-based approaches in clinical practice.

## Conclusion

In this work, we have developed a computationally efficient framework for evaluating key metrics in PBT, including dose delivery, LET and biologically informed metrics such as RBE and cell survival fraction. Leveraging a simple analytical model, we achieve results that show good agreement with those from computationally intensive Monte Carlo particle simulations, while significantly reducing computational cost. This makes the framework particularly well-suited for rapid evaluations in treatment planning.

The speed and simplicity of the approach enable the exploration of optimisation strategies with respect to challenging objectives, such as LET-weighted dose or survival fraction. Such objectives, while important for improving treatment outcomes, would require significant computational resources if approached using Monte Carlo simulations. Our framework allows for the efficient evaluation of these biologically informed metrics, providing a practical tool for exploring their potential integration into treatment planning workflows as well as for investigating the impact of model uncertainty, particularly in scenarios involving OARs.

By presenting these ideas in a mathematically rigorous but approachable way, we believe this work also serves as an accessible introduction for mathematicians interested in contributing to the field of PBT. The integration of physical, biological and computational principles offers a clear pathway for mathematical researchers to engage with and address real-world challenges in cancer therapy.

This work demonstrates the value of computationally fast models in bridging the gap between theoretical modelling and practical application in PBT. We believe it provides a foundation for future investigations which account for other interaction mechanisms, Coulomb and nuclear, into biologically informed treatment planning, enabling the rapid assessment of new metrics and strategies that may otherwise be computationally prohibitive, supporting the broader goal of delivering better patient outcomes in personalised cancer therapies.

While the current framework is developed in a one-dimensional setting, real tumours and organs exhibit complex, non-radially symmetric morphologies, requiring consideration of spatial variations in tissue composition and beam orientation. In a clinical setting, additional challenges such as organ motion and heterogeneous stopping power must be addressed for optimal treatment planning. The approach presented here serves as a computationally efficient baseline for understanding the role of stopping power uncertainties and biologically informed metrics in dose optimisation. Future work will focus on extending this framework to higher-dimensional settings, incorporating patient-specific anatomical structures and more advanced robust optimisation techniques.

## Data Availability

The codebase used to generate the figures in this work is available at https://doi.org/10.5281/zenodo.14179258.

## Footnotes

1 Often denoted as $\alpha$ in the literature, but here denoted *c* to avoid confusion with parameters in the Bragg–Kleeman model.
